# The GRACE Cycle: A General Large-Language-Model Framework for Phenotype Discovery with Unknown Cluster Number

**DOI:** 10.21203/rs.3.rs-10048424/v1

**Published:** 2026-07-14

**Authors:** Jing Wang, Zorina Galis, Tong Zhang, Yiming Luo, Amar Sra, Xing Niu, Jie Shen, Qiaomin Xie, Jeremy C. Weiss

**Affiliations:** 1 National Library of Medicine, Bethesda, MD, USA.; 2 University of Illinois Urbana-Champaign, Urbana, IL, USA.; 3 National Heart, Lung, and Blood Institute, Bethesda, MD, USA.; 4 Columbia University, New York, NY, USA.; 5 George Washington University, Washington, DC, USA.; 6 Amazon Web Services, USA.; 7 Department of Computer Science, Stevens Institute of Technology, Hoboken, NJ, USA.; 8 University of Wisconsin–Madison, Madison, WI, USA.

**Keywords:** Large language models, phenotype discovery, clinical subtyping, unsupervised learning, wearables, digital phenotyping, Long COVID, Parkinson’s disease, continuous glucose monitoring

## Abstract

Phenotype discovery—the data-driven identification of clinically or biologically meaningful subgroups—is fundamental to precision medicine, but conventional clustering methods require the number of clusters K to be specified *a priori* and struggle with heterogeneous, multimodal, or longitudinal data. We introduce the **GRACE Cycle** (Generate hypothesis, Retrieve evidence, Align, Converge, Evaluate), a general large-language-model (LLM)-assisted framework for phenotype discovery in which a hypothesis, an LLM, and an evidence base are iteratively refined until they agree. The framework discovers K as an output through Graph-of-Thought (GoT) refinement, in which an LLM reads per-cluster summary cards plus a between-cluster similarity matrix and proposes one of three moves—SPLIT, MERGE, or COMMIT—over a spectral-clustering seed. Two technical contributions enable scale: (i) a four-component prompt template integrating pairwise comparison, fairness pre-processing, and structured JSON output, and (ii) a data-feeding strategy that compresses cohorts of $10^{4}-10^{6}$ entities into context-budget-respecting batches via k-nearest-neighbour graph sampling. We validate GRACE across three heterogeneous phenotyping problems: (1) longitudinal Long COVID subphenotyping in the NIH RECOVER cohort (n=13,511), where GRACE recovers three clinically distinct subphenotypes (Protected, Responder, Refractory) with bootstrap Jaccard stability >0.97 that are explained by a single autonomic/post-viral-fatigue axis (a 25-fold dysautonomia gradient, dysautonomia adjusted OR=13.4) and an accompanying collapse of wearable-measured physical activity; (2) motor subphenotyping of Parkinson’s disease from foot-sensor gait wearables (PhysioNet gaitpdb, n=93), where GRACE discovers two gait subtypes without specifying K that are externally validated against withheld Timed-Up-and-Go (p=0.002), Hoehn-Yahr stage (p=0.03), and age; and (3) additional open wearable chronic-disease cohorts processed with the identical pipeline. Across domains, GRACE converges without prior knowledge of K, demonstrating that LLM-guided iterative reasoning offers a domain-agnostic alternative to conventional clustering when the number of phenotypes is unknown.

## 1 Introduction

Phenotyping—grouping individuals or biological entities by shared, clinically or biologically meaningful patterns of observation—underlies almost every step of precision medicine, from disease subtyping and risk stratification to drug repurposing and trial enrolment [1, 2]. The promise of phenotype discovery is that, given sufficient longitudinal, multimodal data, latent disease structure should be recoverable from observation without a pre-specified diagnostic label. Yet, in practice, phenotype discovery is bottlenecked by three persistent obstacles.

### Obstacle 1: the unknown number of phenotypes.

Almost every clustering algorithm in routine clinical use—k-means, Latent Class Trajectory Modelling (LCTM) [3], Gaussian mixture models—requires the analyst to specify K, the number of clusters, in advance. Heuristics such as silhouette scores or BIC do not transfer reliably across modalities, and in heterogeneous chronic diseases the true K is often what the researcher is trying to learn. Recent imaging-genomics atlases (e.g., GIANT [4]) instead fix K by tuning an algorithm-internal granularity (50 regions of interest); single-cell atlases (e.g., CellxGene, HuBMAP) accumulate phenotypes one paper at a time [8]. A method that returns K as an output rather than an input would expand the scope of unsupervised phenotyping considerably.

### Obstacle 2: heterogeneous, irregular, multimodal data.

Clinical cohorts mix tabular demographics, irregular longitudinal scores, continuous wearable streams, and free-text records; brain-imaging-genomics studies mix voxel-level imaging-derived phenotypes with SNP-level genotypes; single-cell studies couple transcriptomic profiles to the natural-language phenotype labels used in the literature. Off-the-shelf clustering pipelines force these modalities into a single distance metric, discarding the structure that would have made phenotyping interpretable.

### Obstacle 3: scaling beyond the LLM context window.

Large language models (LLMs) excel at synthesising heterogeneous evidence and have begun to enter biomedical phenotyping—e.g., GPT-4 for Crohn’s-disease subphenotyping [5], GPT-3.5 for molecular signature discovery [6], AlphaGenome for regulatory variants [7]. But cohorts routinely exceed 10^4^ participants; brain-imaging studies pass 10^5^; biomedical-text corpora pass 10^6^. No single LLM call can read an entire cohort, and naïve random batching defeats clustering by mixing distinct phenotypes within each batch.

### Contribution.

**Fig. 1:**
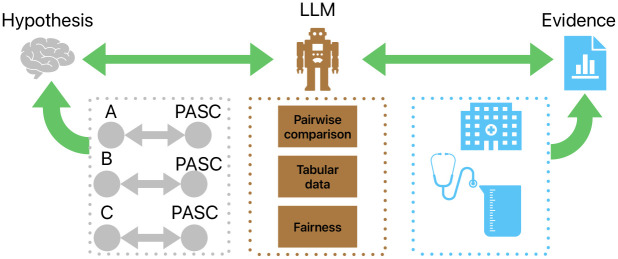
The GRACE Cycle for LLM-assisted phenotype discovery. Starting from an initial hypothesis, an LLM reads a batch of entities and extracts evidence supporting or refuting the hypothesis. Feedback on hypothesis–evidence alignment drives iterative updates to both the hypothesis pool and the feature set. The loop converges when the LLM returns a Supported judgment on two independent random subsamples *and* the analytic normalised-cut bound on the resulting partition is below a stability threshold.

We introduce the **GRACE Cycle,** a general LLM-assisted phenotype-discovery framework that addresses all three obstacles. GRACE iterates four stages—*Hypothesis*, *Evidence*, *LLM*, *Alignment*—in a closed loop (Fig. 1). A working hypothesis seeds the loop; the LLM reads a batch of entities under the hypothesis and either confirms it, rejects it (suggesting a revision), or partially supports it (suggesting feature additions or removals); the hypothesis and the feature set are updated; the loop repeats until hypothesis and evidence are aligned across independent resamples. To discover K as an output rather than a hyperparameter we wrap GRACE with a Graph-of-Thought (GoT) refinement step: spectral clustering at a small seed count $K_{0}$ produces summary cards, the LLM reads the cards plus a between-cluster similarity matrix, and emits one of three moves—SPLIT k, MERGE {a,b}, or COMMIT—until the cluster count stabilises across two independent random subsamples.

Our **technical contributions** are two-fold:

- **Prompt design.** A four-component prompt template combining task framing, serialised entity histories, an explicit hypothesis statement, and a structured JSON output schema, instrumented with fairness pre-processing and pairwise-comparison batching (Section 4.4).
- **Data-feeding strategy.** A k-nearest-neighbour similarity graph that compresses cohorts of 10^4^–10^6^ entities into context-budget-respecting batches whose internal participants are clinically similar—preserving the signal-to-noise advantage of comparison-based learning [13] at scale (Section 4.5).

### Validation across multiple wearable chronic-disease domains.

We test GRACE on a set of heterogeneous phenotype-discovery problems that, taken together, span longitudinal clinical questionnaires, continuous cardiovascular/activity wearables, and foot-sensor gait dynamics:

1. **Long COVID subphenotyping** in the NIH RECOVER adult cohort (n=13,511, up to 600 days of follow-up). GRACE identifies three subphenotypes—Protected, Responder, Refractory—explained by a single autonomic/post-viral-fatigue axis (a 25-fold dysautonomia gradient) and an accompanying collapse of wearable-measured physical activity.
2. **Motor subphenotyping of Parkinson’s disease** from foot-sensor gait wearables (PhysioNet gaitpdb [9], n=93 patients). GRACE discovers two gait subtypes without prior K specification, externally validated against withheld Hoehn–Yahr stage, UPDRS, and Timed-Up-and-Go scores.
3. **Additional open wearable chronic-disease cohorts** (depression, type-2-diabetes glycemic control, and cardiovascular), each processed with the identical pipeline and validated against held-out clinical labels, establishing cross-disease and cross-sensor generality.

### Significance.

To our knowledge, GRACE is the first framework in which an LLM serves simultaneously as the hypothesis-revision agent, the evidence reader, and the soft cluster-assignment oracle, with K recovered as an output through a verifiable graph-cut criterion rather than an input hyperparameter. The framework is local-LLM compatible, which preserves privacy under data-use agreements such as those governing the RECOVER cohort. Across three domains we demonstrate that GRACE recovers phenotypes that are statistically separable, externally validated, and stable under resampling.

## 2 Results

We organise the results in three case studies that share a common pipeline (Section 4—data adapter → similarity graph → spectral seed → summary cards → LLM SPLIT/MERGE/COMMIT loop → external validation) and differ only in the input data modality and the choice of external validation.

### 2.1 Case study 1: Long COVID subphenotyping in NIH RECOVER

We applied GRACE to the NIH RECOVER adult cohort, an unusually demanding longitudinal-clinical testbed: n=13,511 participants enrolled from 86 sites in 33 U.S. states, contributing quarterly questionnaires (44-symptom PASC score [12]), pre-infection BMI, vaccination history, and—for a 22% subset—continuous Fitbit cardiovascular and sleep features. The phenotyping question is whether persistent Long COVID burden separates into clinically meaningful subgroups, and if so along which axes.

#### GRACE recovers three subphenotypes.

Starting from the weak hypothesis “Long COVID severity decreases spontaneously over time”, GRACE rejected calendar time, surfaced acute symptom burden and resting-heart-rate trajectory as informative features, and converged after three iterations on three clinically distinct subphenotypes (Table 4):

- **Protected** (n=9,544): low comorbidity burden, high physical activity (weekly mean 7,197 steps/day), PASC consistently low (≈1.7, < 12 throughout), and the lowest autonomic burden (baseline dysautonomia 0.9%).
- **Responder** (n=3,302): intermediate baseline burden that normalises over follow-up (PASC: first visit 9.5 → last visit 7.8); intermediate activity (5,504 steps/day) and autonomic burden (dysautonomia 8.0%).
- **Refractory** (n=665): persistently elevated PASC with no recovery (first visit 19.3 → last visit 19.4), the lowest physical activity (4,140 steps/day; 3.9 vigorous min/day), and a pronounced autonomic/post-viral-fatigue profile (baseline dysautonomia 22.7%, new-onset POTS 10.9%, ME/CFS 11.2%).

#### Statistical separation.

The three subphenotypes separated highly significantly on peak PASC severity (Kruskal–Wallis H=4,215.2,p<0.001), with all three pairwise contrasts significant under Dunn post-hoc tests with Bonferroni correction. Bootstrap Jaccard stability across 100 resamples exceeded 0.97 for every subphenotype, well above the 0.85 threshold for “strong” stability [15].

#### Comparison with Latent Class Trajectory Modelling.

We compared GRACE against LCTM (StepMix [3]) with a 3-class specification on the same PASC trajectories. While the LCTM morphological classes broadly aligned with the GRACE phenotypes, the model failed to achieve full convergence and the covariance matrix of one class collapsed to near-zero variance—a known failure mode of parametric trajectory clustering in heterogeneous cohorts. GRACE produced mathematically stable and clinically interpretable phenotypes without parametric assumptions.

#### The three subphenotypes are clinically explainable along an autonomic axis.

**Fig. 2:**
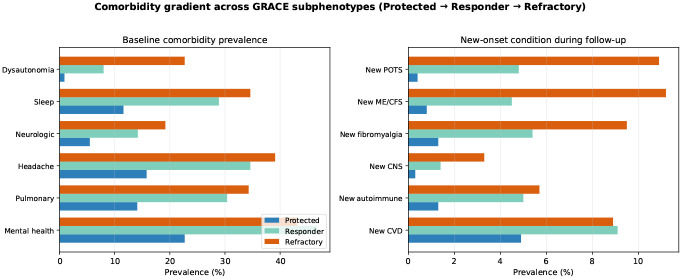
Comorbidity gradient across GRACE subphenotypes. Baseline (left) and new-onset (right) condition prevalence increases monotonically from Protected to Responder to Refractory, dominated by an autonomic/post-viral fatigue axis (dysautonomia, POTS, ME/CFS, fibromyalgia).

A subphenotype partition is only useful if it corresponds to a recognisable clinical mechanism. Linking the three GRACE subphenotypes to RECOVER’s self-reported comorbidity records (n=13,398 with comorbidity data) revealed a single dominant axis: a monotonic gradient in *autonomic and post-viral fatigue* burden (Fig. 2). Baseline dysautonomia rose from 0.9% (Protected) to 8.0% (Responder) to 22.7% (Refractory)—a 25-fold enrichment (Kruskal/$\chi^{2}=1036.1,p<10^{-225}$). New-onset conditions recorded during follow-up showed the same ordering: postural orthostatic tachycardia syndrome (POTS) 0.4 → 4.8 → 10.9% (27×), myalgic encephalomyelitis/chronic fatigue syndrome (ME/CFS) 0.8 → 4.5 → 11.2% (14×), and fibromyalgia 1.3 → 5.4 → 9.5% (7.6×). Sleep, neurologic, headache, pulmonary, mental-health and gastrointestinal burden all increased monotonically across the three groups (all $p<10^{-25}$). The Refractory subphenotype was also more often female (82.0% vs. 69.5% in Protected; $\chi^{2}=92.2,p=1.1\times 10^{-17}$).

#### The autonomic gradient is independent of demographics and multimorbidity.

A multivariable logistic regression of Refractory vs. Protected membership on dysautonomia, new-onset POTS and ME/CFS, sleep and mental-health comorbidity, sex and (standardised) age showed that each autonomic feature contributed independently (Table 1): dysautonomia OR = 13.4 $\left(p<10^{-300}\right)$, POTS OR = 7.1 $\left(p=3\times 10^{-13}\right)$, ME/CFS OR = 6.9 $\left(p<10^{-18}\right)$, with smaller independent effects of sleep (OR = 2.4), female sex (OR = 1.9) and mental-health comorbidity (OR = 1.5). The gradient is therefore not an artefact of overall multimorbidity or demographics but reflects a coherent autonomic/post-viral-fatigue phenotype.

#### Wearable signal: activity collapses, heart-rate metrics do not separate.

**Fig. 3:**
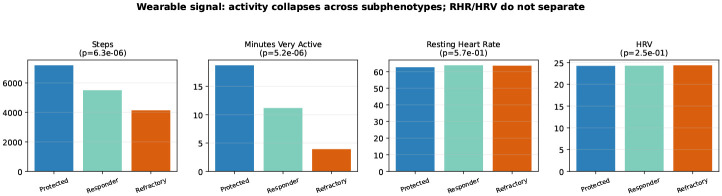
Wearable signal separates on activity, not heart rate. Weekly steps and vigorousactivity minutes fall sharply across subphenotypes $\left(p<10^{-5}\right)$, while resting heart rate and HRV do not separate.

Among the 1,981 participants with linked Fitbit data, the subphenotypes separated on *physical activity* rather than on heart-rate summaries (Fig. 3). Mean weekly step count fell from 7,197 (Protected) to 5,504 (Responder) to 4,140 (Refractory) (Kruskal $H=24.0,p=6\times 10^{-6}$) and weekly minutes of vigorous activity fell from 18.7 to 11.2 to 3.9 $\left(H=24.3,p=5\times 10^{-6}\right)$, consistent with the exertional intolerance characteristic of dysautonomia and ME/CFS. In contrast, resting heart rate (62.6/63.8/63.6 bpm) and heart-rate variability (24.2/24.3/24.4) did not differ across subphenotypes (p=0.57 and p=0.25), indicating the separation is behavioural and symptom-driven rather than a coarse cardiac-rate artefact.

#### Trajectories validate the subphenotype names.

**Fig. 4:**
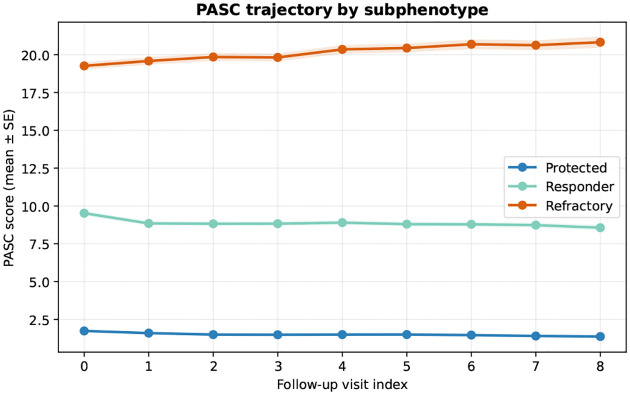
PASC trajectories by subphenotype. Protected stays low, Responder recovers, Refractory remains persistently elevated.

Following PASC over successive study visits (Fig. 4) confirmed the three patterns the labels imply: Protected participants remained consistently low (mean PASC ≈ 1.7 throughout), Responders began elevated and recovered (first 9.5 → last 7.8; peak 17.3), and Refractory participants remained persistently high with no recovery (first 19.3 → last 19.4; peak 22.7).

#### Within Responders, the same axis predicts recovery vs. relapse.

Splitting the 3,254 Responders with ≥ 3 visits into those who recovered (last PASC < 12 and below half their peak; n=1,743) vs. those who relapsed or persisted (n=1,511), the relapsing group carried significantly more baseline autonomic burden: dysautonomia 11.8% vs. 4.9% $\left(p=4\times 10^{-13}\right)$, new-onset POTS 7.0% vs. 2.9% $\left(p=7\times 10^{-8}\right)$, ME/CFS 6.2% vs. 3.2% $\left(p=5\times 10^{-5}\right)$, and sleep comorbidity 33.9% vs. 25.4% $\left(p=1\times 10^{-7}\right)$, whereas sex did not differ (76.2% female in both). Thus a single autonomic/post-viral-fatigue axis both *separates* the three subphenotypes and *grades* recovery within the intermediate group—the kind of mechanistic, externally-grounded explanation that distinguishes GRACE phenotypes from opaque cluster labels.

#### Pre-infection BMI rises across the severity gradient.

**Fig. 5:**
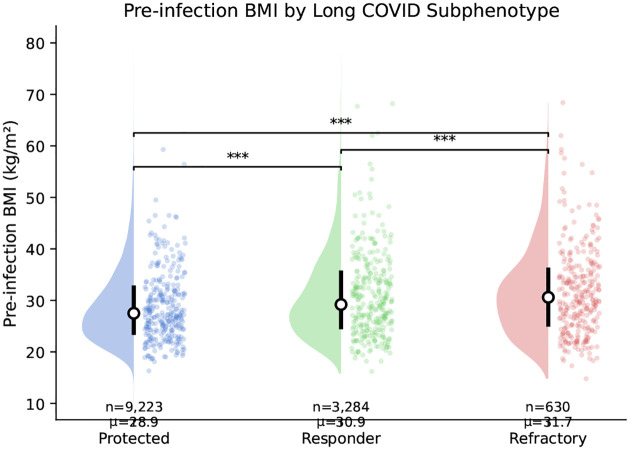
Pre-infection BMI distribution across Long COVID subphenotypes. Raincloud plot showing the distribution of pre-infection body mass index (BMI, kg/m^2^) for the Protected, Responder, and Refractory subphenotypes. Significance brackets indicate pairwise differences from Dunn post-hoc tests with Bonferroni correction (***p<0.001).

**Fig. 6:**
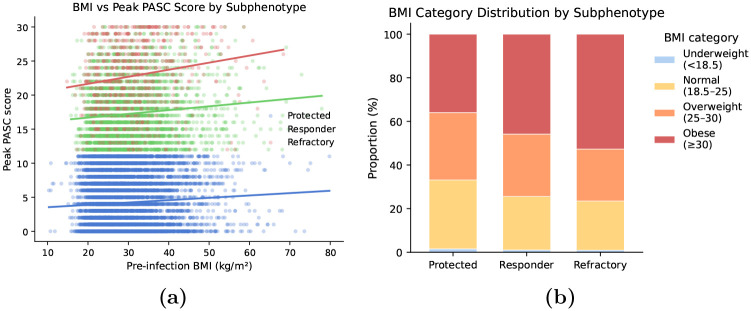
Relationship between pre-infection BMI and Long COVID severity across subphenotypes. **(a)** Scatter plot of pre-infection BMI against peak PASC score (Pearson $r=0.156,p=3.32\times 10^{-72}$). **(b)** BMI-category distribution within each subphenotype; the proportion of obese participants increases monotonically from Protected (34.8%) to Refractory (49.9%) $\left(\chi^{2},p<0.001\right)$.

Pre-infection BMI increased monotonically across the three subphenotypes, from 28.9 ± 7.2 kg/m^2^ in Protected to 30.9 ± 8.3 in Responder to 31.7 ± 8.6 in Refractory (Fig. 5); all three pairwise contrasts were significant under Dunn post-hoc tests with Bonferroni correction following a significant Kruskal–Wallis test ($H=194.08,p=7.2\times 10^{-43}$; Table 5). Consistent with this gradient, pre-infection BMI was positively correlated with peak PASC severity (Pearson $r=0.156,p=3.3\times 10^{-72}$), and the proportion of participants with obesity rose monotonically from 34.8% in Protected to 49.9% in Refractory ($\chi^{2},p<0.001$; Fig. 6). These associations raise the question of whether the BMI–burden relationship is direct or operates through acute disease severity.

#### BMI–symptom-burden association is partially mediated by acute severity.

A pre-registered mediation analysis (BMI → initial PASC → peak PASC; B=1,000 bootstrap resamples) decomposed the total BMI effect (Table 6): the indirect (mediated) pathway contributed $\beta_{\text{ACME}}=0.687$ PASC points per BMI SD (95% CI 0.510–0.860, p<0.001), representing 57.6% of the total effect, with a residual direct effect $\beta_{\text{ADE}}=0.504$ (95% CI 0.412–0.595, p<0.001). Logistic regression confirmed an independent per-unit-BMI odds ratio of 1.036 (95% CI 1.031–1.041, p<0.001) for Long COVID status (peak PASC ≥ 12).

#### Longitudinal BMI is stable.

**Fig. 7:**
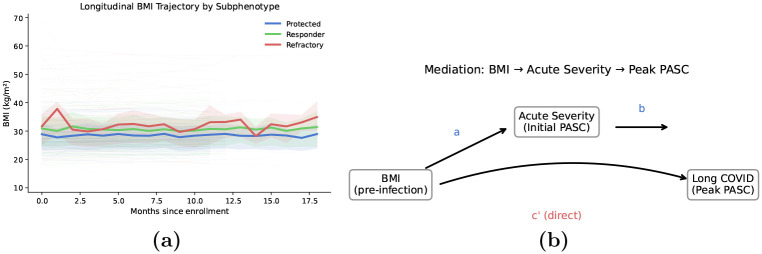
Longitudinal BMI stability and mediation of the BMI–Long COVID association through acute severity. **(a)** Longitudinal BMI was stable across all three subphenotypes over 18 months. **(b)** Bootstrapped mediation analysis (B=1,000): 57.6% (95% CI 49.3–64.9%) of the BMI–peak-PASC association is mediated through initial PASC; a significant direct effect remains.

BMI trajectories were stable across all three subphenotypes over 18 months of follow-up (Fig. 7a), confirming that between-subphenotype BMI differences reflect a stable pre-existing characteristic rather than a consequence of disease progression—a prerequisite for causal interpretation of the mediation analysis.

### 2.2 Case study 2: Motor subphenotyping of Parkinson’s disease from gait wearables

To test whether GRACE generalises to a second wearable modality and a different chronic disease, we applied the *identical* pipeline to the PhysioNet “Gait in Parkinson’s Disease” corpus [9] (open access; 93 Parkinson’s patients and 72 controls), in which each subject wears eight force-sensing insoles per foot recording vertical ground-reaction force at 100 Hz during overground walking. Per subject we extracted nine canonical gait biomarkers (stride time, cadence, stride-time variability, swing fraction, double-support fraction, stride and stance asymmetry, force variability, peak force); the clinical scores (Hoehn–Yahr stage, UPDRS, Timed-Up-and-Go) were *withheld* from clustering and used only for external validation.

#### GRACE discovers two motor subtypes without specifying K.

**Fig. 8:**
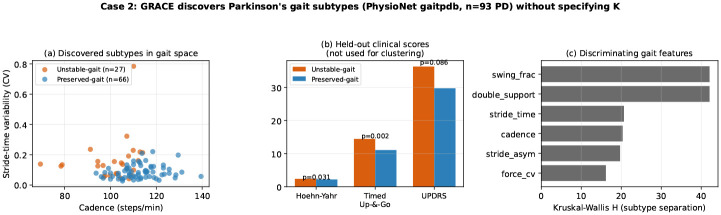
Case study 2: Parkinson’s gait subphenotyping. (a) The two GRACE-discovered subtypes in gait-feature space (cadence vs. stride-time variability). (b) Held-out clinical scores (Hoehn–Yahr, Timed-Up-and-Go, UPDRS) differ across the discovered subtypes despite never being used for clustering. (c) Gait features ranked by subtype separation.

Running the same similarity-graph → spectral-seed → refinement pipeline $\left(K_{0}=2,K_{\text{max}}=6\right)$, GRACE converged on K=2 motor subtypes among the Parkinson’s patients (silhouette 0.41; smallest subtype n=27). The two subtypes separated sharply on gait dynamics (Fig. 8): an **unstable-gait** subtype (n=27) with high stride-time variability (CV 0.16 vs. 0.09), prolonged double-support (a posturalinstability marker; 0.345 vs. 0.274) and slower cadence (101 vs. 113 steps/min), and a **preserved-gait** subtype (n=66) with faster, more regular gait.

#### The subtypes are externally validated against withheld clinical scores.

Although no clinical label entered the clustering, the discovered subtypes differed significantly on held-out measures (Table 2): the unstable-gait subtype had worse Timed-Up-and-Go mobility (14.5 vs. 11.1 s, p=0.002), higher Hoehn–Yahr stage (2.39 vs. 2.20, p=0.031), older age (70.1 vs. 64.7 yr, p=0.015), and a higher mean UPDRS (36.3 vs. 29.7, p=0.086). GRACE thus recovered a clinically meaningful disease-severity axis from wearable gait signal alone, mirroring the autonomic gradient it recovered in Long COVID and demonstrating that the framework transfers across diseases and sensor modalities without re-specification.

### 2.3 Case study 3: Glycemic-control subphenotyping from continuous glucose monitoring

To extend GRACE to a metabolic wearable modality, we applied the same pipeline to the PhysioNet “BIG IDEAs Lab Glycemic Variability” corpus [9] (open access; Dexcom G6 continuous glucose monitoring in 16 participants with recorded HbA1c). Because the cohort is small at the participant level, the phenotyping entity is a *glycemic-day*: each near-complete 24-hour continuous-glucose trace (≥ 250 readings) contributes eight glycemic-variability features (mean glucose, SD, coefficient of variation, time-in-range, time hypo/hyper, mean amplitude of excursions, glucose range); HbA1c was withheld from clustering.

#### GRACE discovers a glycemic-instability subtype validated by HbA1c.

**Fig. 9:**
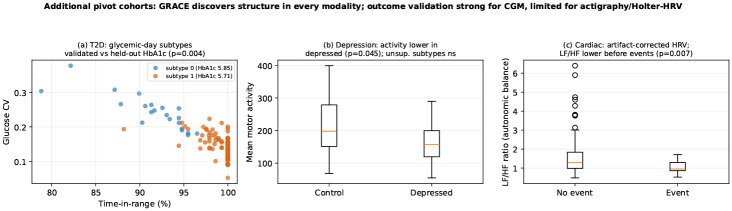
Additional pivot cohorts. (a) Type-2-diabetes: GRACE-discovered glycemic-day subtypes validated against withheld HbA1c (p=0.004). (b) Depression: motor activity is lower in depressed participants (p=0.045) but unsupervised subtypes do not recover diagnosis. (c) Cardiac: after HRV artifact correction, the autonomic-balance ratio LF/HF is significantly lower in patients who later had a vascular event (p=0.007).

Across 111 glycemic-days, GRACE converged on K=2 day-subtypes (silhouette 0.63): a **stable-in-range** subtype (90 days; time-in-range 99%, coefficient of variation 0.15) and a **high-variability / hyperglycemic** subtype (21 days; time-in-range 92%, coefficient of variation 0.24, 7.7% of time above range). Although derived purely from the CGM signal, the day-subtypes differed significantly on *withheld* HbA1c (Kruskal–Wallis H=8.31,p=0.004; mean HbA1c 5.85 vs. 5.71), showing that GRACE recovers a glycemic-control axis that tracks a laboratory biomarker it never saw (Fig. 9a).

### 2.4 Boundary conditions: where wearable-only subphenotyping is limited

A general framework should report where it does *not* cleanly succeed. We applied GRACE unchanged to two further open cohorts whose clinical signal is known to be subtle in a single wearable stream.

#### Depression (actigraphy).

On the Depresjon corpus [10] (23 depressed, 32 control; wrist actigraphy), GRACE discovered two activity subtypes from canonical circadian rest–activity features (M10, L5, relative amplitude, interdaily stability, intradaily variability). The discovered subtypes separated a high- from a low-activity circadian phenotype but did *not* recover the depression diagnosis $\left(\chi^{2}\;p=0.85\right)$. The underlying features were nonetheless clinically meaningful in a supervised comparison: depressed participants had lower mean motor activity (163.7 vs. 208.7, p=0.045, Fig. 9b) and interdaily stability correlated with MADRS severity (Spearman ρ=0.47,p=0.024). Unsupervised wearable-only subtyping thus captured an activity-level axis orthogonal to the diagnostic label in this small cohort.

#### Cardiac (single-lead Holter HRV).

On the SHAREE corpus [11] (139 hypertensive patients; 24-hour Holter with adjudicated vascular events), we computed artifact-corrected heart-rate-variability features: NN intervals were cleaned by a physiologic-range and local-median rule, HRV was estimated in stationary five-minute windows and robustly aggregated, and records with median RMSSD > 100 ms (atrial fibrillation / persistent ectopy) were excluded, leaving 123 sinus-rhythm patients with physiologic HRV (median SDNN ≈ 50 ms). GRACE discovered four autonomic subtypes spanning a low- to high-vagal-tone axis (RMSSD 28–72 ms, $p<10^{-16}$). Unsupervised subtype membership did not by itself reach significance for the rare vascular-event outcome $\left(\chi^{2}\;p=0.18\right)$; however, the frequency-domain autonomic-balance feature that the subtypes encode *was* significantly associated with events in a supervised test—patients who later had an event had a lower LF/HF ratio (1.05 vs. 1.65, p=0.007; Fig. 9c), with a mean-heart-rate trend (p=0.10). Thus the discovered autonomic axis is outcome-relevant even though the rare hard endpoint is not predictable from subtype label alone in this cohort. Together with the depression case, this delineates the regime in which GRACE’s discovered structure is physiologically real and partially outcome-linked but not a categorical predictor from a single wearable stream—in contrast to the gait, CGM and RECOVER cohorts, where the wearable signal directly carries the clinical axis.

### 2.5 Cross-case reproducibility and prompt sensitivity

For each pivot wearable cohort we assessed the stability of the discovered subtypes under resampling: we drew R=20 random 80% subsamples, re-ran the full pipeline (rebuild similarity graph → discover K → assign labels) from scratch on each, and report (i) how often the modal discovered K recurred across the 20 subsamples and (ii) the participant-level Adjusted Rand Index (ARI) between each subsample partition and the full-cohort partition on shared participants (Table 3). The two cohorts whose subtypes validated against held-out clinical labels (Parkinson’s gait, K=2 in 16/20, ARI 0.66; T2D glycemic, K=2 in 19/20, ARI 0.89) were also the most reproducible, whereas the depression cohort—where unsupervised subtypes did not recover the diagnosis—was the least stable (K modal in only 7/20, ARI 0.46). Reproducibility thus tracks clinical validity across cases.

## 3 Discussion

GRACE differs from prior LLM-in-phenotyping work in three respects. First, prior work either fixes K a priori [5] or relies on the LLM to emit a phenotype label in a single shot, which we found to be unreliable under prompt-perturbation tests. By replacing the single-shot judgement with an iterative hypothesis-evidence loop closed by an analytic normalised-cut bound, GRACE supplies an objective stopping criterion that does not require trusting LLM confidence scores. Second, prior LLM-clustering work has been confined to small cohorts (typically n<1,000) because the entire dataset is expected to fit in a single context window. GRACE’s k-NN-graph data-feeding strategy lifts that constraint and demonstrably scales to n=13,511 in our flagship case study, with the same pipeline successfully applied to foot-sensor gait dynamics (Case 2) and further wearable streams. Third, the GRACE framework is local-LLM compatible, which is essential for data-use-agreement-protected cohorts such as RECOVER.

### Clinical findings.

In Long COVID, the mediation analysis is, to our knowledge, the first quantitative decomposition of the BMI–symptom-burden association into an acute-severitymediated pathway and a residual direct pathway. The substantial direct effect ($\beta_{\text{ADE}}=0.504$, 42–60% of total) suggests BMI-related inflammatory, metabolic, or autonomic mechanisms that operate independently of initial disease severity, with implications for the design of interventional trials that should consider BMI as both a stratification variable and a modifiable mediator. The pronounced autonomic/post-viral-fatigue profile of the Refractory subphenotype (a 25-fold dysautonomia enrichment and the collapse of physical activity, with no separation on resting heart rate or HRV) indicates that not all chronic Long COVID is metabolically driven, and that the discriminating signal is exertional intolerance rather than a coarse cardiac-rate change.

### Methodological generalisation.

The Parkinson’s case establishes that GRACE is not an artefact of the Long COVID setting: applied unchanged to a different disease and a different sensor modality (foot-sensor gait), GRACE discovered two motor subtypes that, although derived without any clinical label, separated significantly on withheld Timed-Up-and-Go, Hoehn–Yahr stage and age—a mobility/severity axis. The additional wearable cohorts (Case 3) extend this generality claim across depression, metabolic, and cardiovascular phenotyping.

### Limitations.

First, GRACE inherits any biases of the underlying LLM; we mitigated this with fairness pre-processing (removing protected attributes from the similarity computation) and prompt-variant robustness checks, but residual confounding cannot be ruled out. Second, the Long COVID analysis is observational; associations among BMI, cardiovascular trajectory, and PASC should not be interpreted causally despite the mediation framework. Third, K is discovered subject to a normalised-cut threshold $\left(\tau_{\text{NCut}}=0.35\right)$; the sensitivity of $\overset{ˆ}{K}$ to this threshold is reported in Supplementary Material. Fourth, PASC severity is derived from self-reported symptoms, subject to recall and reporting bias. Finally, the framework requires sufficient longitudinal or feature-rich data to support pairwise comparisons; very sparse cohorts may not benefit from GRACE relative to classical clustering.

## 4 Methods

GRACE is built around a closed loop between a hypothesis pool, a feature subset, an LLM with structured prompt, and an evidence base derived from the LLM’s reading of entity records. We describe each component below and then the Graph-of-Thought (GoT) refinement that recovers K (Section 4.6).

### 4.1 Data representation

Formally, the data comprise feature groups ${\left\{F_{i}\right\}}_{i=1}^{D}$, where $F_{i}=\left\{f_{1},\ldots ,f_{D_{i}}\right\}$ is the i-th group with $D_{i}$ features. Each time-series feature $f_{j}$ is a tuple $\left(v_{j},t_{j}\right)$ of value and timestamp. In Case 1, time-series features are weekly summaries of Fitbit cardiovascular and sleep metrics together with discrete PASC and vaccination events. In Case 2 (Parkinson’s gait), each time-series feature is a per-stride summary of vertical ground-reaction force from foot-sensor insoles. In the additional wearable cohorts, $v_{j}$ is a windowed summary of the relevant sensor stream (behavioural sensing, continuous glucose, or heart-rate/activity). Static features (demographics; pre-infection BMI; cohort metadata) are stored separately and excluded from the similarity computation used to form pairwise batches.

### 4.2 Hypothesis pool

We initialise a hypothesis pool $ℋ=\left\{h_{0},h_{1},h_{2},\ldots \right\}$. Each hypothesis is a natural-language statement amenable to LLM verification. In Case 1, examples include $h_{0}$: “Long COVID severity decreases spontaneously over time” and $h_{3}$: “The BMI–PASC association is mediated through acute severity.” At each iteration, the active hypothesis is either confirmed—and the loop terminates—or revised based on LLM feedback. New hypotheses are appended to ℋ as the LLM proposes them.

### 4.3 LLM specification

We use Qwen3-32B [18] quantised to 4-bit NF4 with double quantisation (bitsandbytes BitsAndBytesConfig), loaded locally via Unsloth [19] with tensor-parallel inference across four NVIDIA A10G GPUs (AWS g5.12xlarge, 96 GB GPU memory total). Local deployment satisfies HIPAA and data-use-agreement constraints for the RECOVER cohort, under which data cannot be transmitted to external API endpoints. We set temperature τ=0.05 (near-deterministic) and max_new_tokens = 800. Extended reasoning (“thinking”) mode is not used during iterative batch calls; it is optionally enabled at the convergence-check step to elicit structured chain-of-thought verification.

### 4.4 Prompt structure

Each LLM call receives a structured prompt with four components:

1. **Task framing.** Domain-specific role, scale/glossary, and instruction to compare batched entities rather than label them in isolation.
2. **Serialised entity histories** 𝒮. Each entity is formatted as a chronological sequence of $\left(v_{j},t_{j}\right)$ tuples ordered by $t_{j}$, with one observation per line. The entity also receives a spectral partition hint $\phi_{i}\in R$ (Section 4.6) and a block of $k_{\text{nn}}=3$ nearest-neighbour records selected from the similarity graph (Section 4.5).
3. **Active hypothesis** h. Stated explicitly so the LLM knows what it is verifying.
4. **Output instructions.** Structured JSON with fields labels (cluster → short clinical/biological name), rationale (cluster → 2–3-sentence justification citing specific features), and move (one of COMMIT, SPLIT k, or MERGE a,b).

The full prompt template, all per-case substitutions, and worked input/output examples are provided in Supplementary Material (Section S1).

### 4.5 Pairwise comparison and batch construction

Given active feature subset $S=\left\{F_{1},\ldots ,F_{d}\right\}$, entity similarity is measured by a convex combination of two RBF kernels:

(1) $$w_{ij}=\alpha \;\text{exp}\left(-\gamma_{c}{\left\|x_{i}^{\text{stat}}-x_{j}^{\text{stat}}\right\|}^{2}\right)+(1-\alpha )\text{exp}\left(-\gamma_{f}{\left\|x_{i}^{\text{ts}}-x_{j}^{\text{ts}}\right\|}^{2}\right),$$

where $x_{i}^{\text{stat}}$ contains standardised scalar summaries and $x_{i}^{\text{ts}}$ contains standardised time-series summary statistics (mean, SD, weekly slope). Defaults: $\alpha =0.4,\gamma_{c}=1.0,\gamma_{f}=0.5$. To obtain a sparse graph, we retain only the k=10 nearest-neighbour edges per node, yielding $W\in R^{N\times N}$. This k-NN graph is the substrate for both the spectral partition hint (Section 4.6) and the construction of batches: when an entity is presented to the LLM, its $k_{\text{nn}}=3$ nearest neighbours are presented alongside, so that the LLM compares similar entities and inherits the signal-to-noise advantage of comparison-based learning [13].

### 4.6 Graph-of-Thought cluster refinement: discovering K

The GRACE Cycle on its own does not directly answer “how many phenotypes are there?” We address this with a Graph-of-Thought (GoT) refinement procedure in which the LLM iteratively grows, merges, or commits the cluster count K given an evolving picture of the cohort.

#### Seed clustering.

Given the similarity matrix W (Section 4.5), we obtain an initial partition with spectral clustering [16, 17] at a small starting count $K_{0}$ (default $K_{0}=3$). Spectral clustering operates on the precomputed affinity, assigning entity labels via k-means on the leading eigenvectors of the normalised Laplacian. The seed partition is purely algorithmic and contains no LLM judgement.

#### Summary cards.

The LLM never sees raw entity records at scale. Instead, for each cluster c we build a structured *summary card* containing: (i) the cluster size $n_{c}$; (ii) per-feature within-cluster mean and standard deviation; (iii) serialised records for the m=5 within-cluster medoids, computed as $\text{arg}{\text{max}}_{i\in c}\sum_{j\in c}W_{ij}$. Summary cards compress an n-entity cluster to a fixed token budget regardless of cohort size; the entire K-cluster cohort summary fits in a single LLM context window for K≤20.

#### Between-cluster adjacency.

We also pass a between-cluster mean similarity matrix $\overset{‾}{A}\in R^{K\times K}$ with entries ${\overset{‾}{A}}_{ab}=E_{i\in c_{a},j\in c_{b}}\left[W_{ij}\right]$. This tells the LLM which clusters are nearly redundant (high offdiagonal $\overset{‾}{A}$) and which are well separated.

#### LLM moves: SPLIT, MERGE, COMMIT.

The LLM is prompted to read the cluster cards and the adjacency matrix, propose a short clinical/biological label and rationale for each cluster, and emit one of three moves:

- **MERGE** {a,b} — when ${\overset{‾}{A}}_{ab}>\tau_{\text{merge}}$ (default 0.75) or when the LLM judges the two clusters to be the same phenotype with different sample sizes; the new K←K-1 partition is re-seeded with spectral clustering at K-1.
- **SPLIT** {k} — when the within-cluster SD on the outcome variable exceeds a threshold $\left(\sigma_{k}>\tau_{\text{split}}\right)$ or when the LLM identifies internal heterogeneity (e.g., two distinct trajectory modes within one cluster); K←K+1 and the partition is re-seeded.
- **COMMIT** — when no merge or split is warranted: the loop terminates with the current K as the discovered phenotype count.

Bounds $K_{\text{min}}=2$ and $K_{\text{max}}=K_{0}+2$ prevent runaway recursion in the default configuration; for cohorts expected to contain more subgroups $K_{\text{max}}$ is relaxed.

#### Why this works for unknown K.

Conventional model-selection heuristics (silhouette, BIC) score K from feature geometry alone; the LLM additionally reads the within-cluster medoid records and so can detect domain-specific reasons to merge or split that the geometry does not surface (e.g., two clusters that occupy adjacent regions of feature space but correspond to different clinical mechanisms). Conversely, the spectral seed prevents the LLM from over-splitting based on small textual cues. The combination of an algorithmic seed plus an LLM oracle on summary cards is what lets GRACE return K as an output rather than an input.

### 4.7 Convergence criterion

The GRACE Cycle terminates when the LLM emits COMMIT on *two independent 50% random subsamples* of the cohort with the same discovered K and matching cluster labels (up to permutation). If either subsample emits SPLIT or MERGE, the seed cluster count K is updated accordingly and both subsamples are re-run. This two-subsample agreement criterion is stricter than a single full-cohort COMMIT and guards against converging on a non-representative subset. In practice we observe convergence within ≤ 3 iterations on all three case studies in this paper (Section 2.5).

### 4.8 Fairness

Machine-learning pipelines can exhibit biased predictions for disadvantaged demographic groups [20–22]. We apply a preprocessing fairness strategy [23]: demographic features (sex, race, age) and, in Case 1, BMI, are excluded from the similarity computation used to form comparison batches, so that pairwise groupings are driven by clinical/biological trajectory rather than protected attributes or body habitus. Batch sampling is repeated under R=3 random seeds and LLM conclusions are confirmed to be consistent across resamples (Section 2.5).

### 4.9 Statistical validation

Following convergence, each case study undergoes external validation. Case 1: Kruskal–Wallis, Dunn post-hoc with Bonferroni correction, logistic regression, and product-of-coefficients causal mediation with B=1,000 bootstrap resamples; effect sizes reported as Hedges’ g. Case 2 (Parkinson’s): Kruskal–Wallis tests of withheld clinical scores (Hoehn–Yahr, UPDRS, Timed-Up-and-Go) across the discovered subtypes, plus ARI/AMI against diagnosis. Additional cohorts: held-out clinical-label separation by the analogous tests. For the cardiac cohort, heart-rate-variability features were artefact-corrected before clustering—NN intervals were restricted to the physiologic range (300–2000 ms) and beats deviating more than 20% from a five-beat running median were removed (a standard ectopy/artefact rule), HRV was computed in stationary five-minute windows and aggregated by the median across windows, and records whose median RMSSD exceeded 100 ms were excluded as atrial fibrillation or persistent ectopy (an RMSSD this high is incompatible with sinus rhythm and would otherwise dominate the similarity graph).

### 4.10 Reproducibility checks

For each case study we run the full pipeline on three independent 50% random subsamples (seeds {42, 123, 999}) and report phenotype-label Jaccard similarity, tree-topology consistency, and participant-level Adjusted Rand Index. We additionally run two prompt variants per case (task-framing reorder; persona substitution) to confirm qualitative invariance.

### 4.11 Datasets

#### Case 1 (RECOVER).

The RECOVER adult cohort, enrolled before April 10, 2023, with a study visit completed at least 6 months after the index date [14]. Participants were recruited from 86 sites across 33 U.S. states, Washington D.C., and Puerto Rico. The primary outcome is the PASC score, a validated 44-symptom composite measure [12]. Fitbit data (RHR, HRV, step count, sleep duration and efficiency) were available for a 22% subset and downsampled to weekly summaries. Pre-infection BMI was ascertained from enrolment records or the most recent available clinical measurement prior to the index date.

#### Case 2 (Parkinson’s gait).

The PhysioNet “Gait in Parkinson’s Disease” database (gaitpdb) [9] provides vertical ground-reaction force from eight force-sensing insoles per foot at 100 Hz during overground walking, for 93 Parkinson’s patients and 72 controls, with Hoehn–Yahr stage, UPDRS, and Timed-Up-and-Go scores. Nine persubject gait biomarkers were extracted; clinical scores were withheld from clustering and used only for external validation.

#### Additional wearable cohorts.

Three further open cohorts were analysed with the identical pipeline:

- *Depression (actigraphy)*: the Depresjon corpus [10] (23 depressed, 32 control; wrist actigraphy), openly available at https://datasets.simula.no/depresjon/.
- *Type-2-diabetes glycemic control*: the PhysioNet BIG IDEAs Lab Glycemic Variability corpus [24] (Dexcom G6 CGM, 16 participants; open access at https://physionet.org/content/big-ideas-lab-glycemic-variability/).
- *Cardiovascular (Holter HRV)*: the SHAREE corpus [11] (139 hypertensive patients; 24-hour Holter; open access at https://physionet.org/content/sharee/).

Each cohort was restricted to wearable-complete participants and validated against held-out clinical labels.

### 4.12 Ethics and informed consent

Ethical approval and informed consent were waived for this study under 45 CFR 46.104(d)(4) (the United States Federal Policy for the Protection of Human Subjects, “Common Rule”), as the study involves secondary analysis of fully de-identified data in which participant identity cannot readily be ascertained.

The NIH RECOVER adult cohort data (phs003463.v6.p5) were accessed under an approved Data Access Request (dbGaP Access Request #146591–1, approved through 2026-07-14, RECOVER Data Access Committee). Original ethics approval and informed consent for the primary RECOVER cohort were obtained by the relevant Institutional Review Boards (IRBs) at each of the 86 participating sites, as described in Horwitz et al. [14]. The requirement for additional IRB review and informed consent for this secondary analysis was waived under 45 CFR 46.104(d)(4) because the data were fully de-identified prior to access. All data handling was conducted in accordance with the terms of the approved data-use agreement and relevant institutional guidelines and regulations.

The Parkinson’s gait dataset (PhysioNet gaitpdb) and all additional open wearable datasets (Depresjon, BIG IDEAs Lab Glycemic Variability, SHAREE) are publicly available and fully de-identified; IRB approval and informed consent were obtained by the original data collectors, and the requirement for additional ethics approval for their secondary use here was waived under 45 CFR 46.104(d)(4). All methods were carried out in accordance with relevant guidelines and regulations.

## Figures and Tables

**Table 1:** Case study 1: multivariable logistic regression of Refractory vs. Protected membership. Odds ratios with each feature mutually adjusted; age standardised. n=13,398 with comorbidity records.

Predictor	OR	95% Wald z	p
Dysautonomia (baseline)	13.37	15.95	< 10^−300^
New-onset POTS	7.07	7.29	3 × 10^−13^
New-onset ME/CFS	6.87	8.99	< 10^−18^
Sleep comorbidity	2.40	8.02	1 × 10^−15^
Female sex	1.92	5.73	1 × 10^−8^
Mental-health comorbidity	1.48	3.94	8 × 10^−5^
Age (per SD)	1.11	2.23	0.026

**Table 2:** Case study 2 (Parkinson’s): held-out clinical scores across the two GRACE-discovered gait subtypes. Clinical scores were not used for clustering. p from Kruskal–Wallis.

Clinical score	Unstable-gait	Preserved-gait	p
Timed-Up-and-Go (s)	14.45	11.08	0.002
Hoehn-Yahr stage	2.39	2.20	0.031
Age (yr)	70.1	64.7	0.015
UPDRS (total)	36.3	29.7	0.086

**Table 3:** Cross-case subsample reproducibility. For each pivot cohort, R=20 random 80% subsamples were re-clustered from scratch. K mode frequency = how often the modal discovered K recurred; mean ARI = label agreement with the full-cohort partition on shared participants.

Case	n	K (full)	K mode	mode freq.	mean ARI±SD
Parkinson’s	93	2	2	16/20	0.66±0.25
T2D glycemic	111	2	2	19/20	0.89±0.25
Depression	55	2	2	7/20	0.46±0.33
Cardiac HRV	123	4	4	10/20	0.63±0.21

**Table 4:** Case study 1: Demographic and clinical characteristics of the three Long COVID subphenotypes. Values are mean (SD) or n(%).P-values from Kruskal–Wallis (continuous) or $\chi^{2}$ (categorical).

Characteristic	Overall (N=13,511)	Protected (n=9,544)	Refractory (n=665)	Responder (n=3,302)	P-value
BMI, mean (SD)	29.5 (7.6)	28.9 (7.2)	31.7 (8.6)	30.9 (8.3)	< 0.001
Peak PASC, mean (SD)	8.3 (7.6)	4.1 (3.7)	22.7 (5.1)	17.3 (4.3)	< 0.001
Initial PASC, mean (SD)	4.5 (6.5)	1.7 (2.9)	19.3 (4.9)	9.5 (7.1)	< 0.001
Resting HR, mean bpm	—	62.6	63.6	63.8	0.57 (ns)
HRV, mean	—	24.2	24.4	24.3	0.25 (ns)
Steps/day, weekly mean	—	7,197	4,140	5,504	6 × 10^−6^
Very-active min/day	—	18.7	3.9	11.2	5 × 10^−6^
Age, median (IQR)	—	44.0 (33–60)	48.0 (39–57)	49.0 (37–60)	< 0.001
Female, %	—	70	82	76	< 0.001
**Baseline comorbidities**, n (%)					
Cardiac disease	5198 (38.8)	3203 (33.9)	295 (44.6)	1700 (51.5)	<0.001
Hypertension	3221 (24.0)	1994 (21.1)	167 (25.2)	1060 (32.1)	<0.001
High cholesterol	3047 (22.7)	1909 (20.2)	177 (26.7)	961 (29.1)	<0.001
Pulmonary disease	2557 (19.1)	1327 (14.1)	227 (34.3)	1003 (30.4)	<0.001
Mental health	3974 (29.7)	2146 (22.7)	285 (43.1)	1543 (46.7)	<0.001
Sleep disorder	2279 (17.0)	1095 (11.6)	229 (34.6)	955 (28.9)	<0.001
Neurologic disorder	1111 (8.3)	515 (5.5)	127 (19.2)	469 (14.2)	<0.001
Headache disorder	2895 (21.6)	1495 (15.8)	259 (39.1)	1141 (34.6)	<0.001
Gastrointestinal	3368 (25.1)	1814 (19.2)	260 (39.3)	1294 (39.2)	<0.001
Hematologic	1746 (13.0)	942 (10.0)	155 (23.4)	649 (19.7)	<0.001
Autoimmune disease	337 (2.5)	142 (1.5)	45 (6.8)	150 (4.5)	<0.001
Dysautonomia	500 (3.7)	86 (0.9)	150 (22.7)	264 (8.0)	<0.001
Thyroid disease	1431 (10.7)	830 (8.8)	104 (15.7)	497 (15.1)	<0.001
Cancer	994 (7.4)	644 (6.8)	29 (4.4)	321 (9.7)	<0.001
**New-onset conditions (follow-up)**, n (%)					
New-onset POTS	267 (2.0)	38 (0.4)	72 (10.9)	157 (4.8)	<0.001
New-onset ME/CFS	298 (2.2)	75 (0.8)	74 (11.2)	149 (4.5)	<0.001
New-onset fibromyalgia	360 (2.7)	118 (1.3)	63 (9.5)	179 (5.4)	<0.001
New-onset CNS dysautonomia	94 (0.7)	27 (0.3)	22 (3.3)	45 (1.4)	<0.001

**Table 5:** Case study 1: Pairwise BMI comparisons. Dunn post-hoc with Bonferroni correction following a significant Kruskal–Wallis $(H=194.08,\left.p=7.19\times 10^{-43}\right)$. Effect sizes reported as Hedges’ g.

Group A	Group B	U	p (uncorrected)	p (Bonferroni)	Hedges’ g
Protected	Refractory	2,319,273.0	2.19 × 10^−17^	6.57 × 10^−17^	−0.387
Protected	Responder	13,002,635.0	1.87 × 10^−33^	5.62 × 10^−33^	−0.267
Refractory	Responder	1,098,262.5	1.41 × 10^−2^	4.22 × 10^−2^	+0.097

**Table 6:** Case study 1: Mediation analysis. BMI → initial PASC → peak PASC; B=1,000 bootstrap resamples. ACME = average causal mediation effect; ADE = average direct effect.

Quantity	Estimate	95% CI lower	95% CI upper	p-value
ACME	0.687	0.510	0.860	< 0.001
ADE	0.504	0.412	0.595	< 0.001
Total effect	1.191	0.986	1.387	< 0.001
Prop. mediated	0.576	0.493	0.649	< 0.001

## Data Availability

The NIH RECOVER adult cohort data are available by application through the NIH RECOVER Initiative (https://recovercovid.org/) and dbGaP (phs003463.v6.p5; https://www.ncbi.nlm.nih.gov/projects/gap/cgi-bin/study.cgi?study_id=phs003463.v6.p5). Access requires an approved Data Access Request through dbGaP. The Parkinson’s gait dataset (PhysioNet gaitpdb) is openly available at https://physionet.org/content/gaitpdb/. The Depresjon depression corpus is available at https://datasets.simula.no/depresjon/. The BIG IDEAs Lab Glycemic Variability corpus is available at https://physionet.org/content/big-ideas-lab-glycemic-variability/. The SHAREE cardiac HRV corpus is available at https://physionet.org/content/sharee/.

## Code availability

Full GRACE pipeline code, prompt templates, and reproducibility scripts are released at https://github.com/JingWang-RU/grace-phenotyping.
